# Associations of serum amino acids related to urea cycle with risk of chronic kidney disease in Chinese with type 2 diabetes

**DOI:** 10.3389/fendo.2023.1117308

**Published:** 2023-03-02

**Authors:** Wei Zhang, Jun Zheng, Jikun Zhang, Ninghua Li, Xilin Yang, Zhong-Ze Fang, Qiang Zhang

**Affiliations:** ^1^ Department of Epidemiology and Biostatistics, School of Public Health, Tianjin Medical University, Tianjin, China; ^2^ Department of Geriatrics, Tianjin Medical University General Hospital, Tianjin Geriatrics Institute, Tianjin, China; ^3^ Department of Toxicology and Sanitary Chemistry, School of Public Health, Tianjin Medical University, Tianjin, China

**Keywords:** type 2 diabetes mellitus, chronic kidney disease, serum amino acids, citrulline, urea cycle

## Abstract

**Objective:**

Serum levels of amino acids related to urea cycle are associated with risk of type 2 diabetes mellitus (T2DM). Our study aimed to explore whether serum levels of amino acids related to urea cycle, i.e., arginine, citrulline, and ornithine, are also associated with increased risk of chronic kidney disease (CKD) in T2DM.

**Methods:**

We extracted medical records of 1032 consecutive patients with T2DM from the Electronic Administrative System of Liaoning Medical University First Affiliated Hospital (LMUFAH) system from May 2015 to August 2016. Of them, 855 patients with completed data available were used in the analysis. CKD was defined as estimated glomerular filtration rate (eGFR) <60 mL/min/1.73 m^2^. Serum amino acids were measured by mass spectrometry (MS) technology. Binary logistic regression was performed to obtain odds ratios (ORs) and their 95% confidence intervals (CIs).

**Results:**

52.3% of the 855 T2DM patients were male, and 143 had CKD. In univariable analysis, high serum citrulline, high ratio of arginine to ornithine, and low ratio of ornithine to citrulline were associated with markedly increased risk of CKD (OR of top vs. bottom tertile: 2.87, 95%CI, 1.79-4.62 & 1.98, 95%CI,1.25-3.14 & 2.56, 95%CI, 1.61-4.07, respectively). In multivariable analysis, the ORs of citrulline and ornithine/citrulline ratio for CKD remained significant (OR of top vs. bottom tertile: 2.22, 95%CI, 1.29-3.82 & 2.24, 1.29-3.87, respectively).

**Conclusions:**

In Chinese patients with T2DM, high citrulline and low ornithine/citrulline ratio were associated with increased risk of CKD.

## 1 Introduction

Chronic kidney disease (CKD) is one of the major microvascular complications of type 2 diabetes mellitus (T2DM) (1–3) and a leading cause of end-stage renal disease (ESRD) in patients with T2DM (4). CKD is associated with an increasing risk of adverse outcomes including cardiovascular disease, infection and death, posing a significant threat to healthcare systems and having negative impacts on quality of life (5, 6). Official figures showed that CKD affected between 8% and 16% of the population worldwide and was a leading cause of death (7). A national survey conducted in China in 2012 revealed that the overall prevalence of CKD was 10.8% (10.2–11.3) (8), which indicated that CKD has become an important public health problem in China.

Growing evidence from clinical research suggests that interventions such as glycemic control, blood pressure control, lipid-lowering measures, use of RAS blockers, weight control and lifestyle modifications can delay the progression of CKD, but the residual risk of CKD remains substantial. Therefore, it is essential to explore novel potential biomarkers that can be used to predict CKD in patients with T2DM.

Over recent decades, rapid advances in bioassay technology have led to the detection of a growing number of metabolites, which have provided new insights into pathways and biomarkers related to diabetes and its complications (9–12). It is worth noting that many studies have found that plasma amino acid levels are significantly altered in patients with T2DM or CKD. Amino acids involved in urea cycle might play an important role in inflammatory markers and oxidative stress (13, 14), many researchers took efforts to examine the relationship between such amino acids and T2DM or CKD. The complete urea cycle is mainly expressed in liver and the products and substrates in the cycle include arginine, citrulline and ornithine. An animal experiment showed that abnormal amino acid metabolism in the urea cycle might be associated with insulin resistance (15). Nokhoijav et al. found that glutamine metabolism, urea cycle, and beta-oxidation make up crucial parts of the metabolic changes in T2DM. In addition, it has been demonstrated that CKD mice had high levels of arginine and citrulline (16). Strong evidence supports the hypothesis that citrulline is a possible biomarker of kidney metabolism (17, 18). However, the association between amino acids related to urea cycle and CKD in T2DM was largely unknown.

We conducted a cross-sectional survey in a Chinese population with T2DM to investigate any associations between serum levels of amino acids involved in urea cycle and the risk of CKD.

## 2 Materials and methods

### 2.1 Study design and population

The details of the study population and methods were described previously (19). Briefly, from May 2015 to August 2016, we retrieved the electronic medical records of 1898 consecutive patients with a diagnosis of T2DM in Liaoning Medical University First Affiliated Hospital (LMUFAH), Jinzhou, China. T2DM was diagnosed by the 1999 World Health Organization’s criteria (20) or treated with antidiabetic drugs. A total of 1032 patients had metabolite data and complete data collection on age, gender, and body mass index (BMI). We used the data of 855 patients in the current analysis after excluding those who lacked creatinine or serum albumin information. The LMUFAH Clinical Research Ethics Committee approved the ethics of the study, and informed consent was waived due to the retrospective character of the cross-sectional study, which is consistent with the Helsinki Declaration.

### 2.2 Data collection

Height, weight and blood pressure were measured by experienced physicians and nurses through standardized methods. To measure height and weight, study participants were asked to take off their shoes and heavy clothing. Body mass index (BMI) was calculated as weight in kilogram divided by height in squared meter height. Blood pressure was measured in a relaxed sitting position. Venous blood samples were drawn in the morning after at least 8 hours. High-density lipoprotein cholesterol (HDL-C), low-density lipoprotein cholesterol (LDL-C), triglyceride (TG), glycated hemoglobin (HbA1c), serum albumin (Alb), serum urea nitrogen (SUN) and serum creatinine (Scr) were assayed in the central laboratory of the hospital. Demographic, lifestyle information and clinical data were also documented, including age, gender, duration of diabetes, smoking status, alcohol consumption, diabetic retinopathy (DR), coronary heart disease (CHD), stroke, use of anti-diabetes drugs, lipid-lowering drugs and antihypertensive drugs.

### 2.3 Clinical definitions

BMI was classified into four categories: underweight (<18.5 kg/m^2^), normal weight (18.5-23.9 kg/m^2^), overweight (24-27.9 kg/m^2^) and obesity (≥28 kg/m^2^) as recommended by the National Health Commission in China (21). According to the criteria recommended by the American Diabetes Association (7), hyperglycemia was defined as HbA1c>7.0%, and dyslipidemia was defined as TG≥1.7mmol/L, LDL-C ≥2.6mmol/L or HDL-C ≤1mmol/L in men and HDL-C ≤1.3mmol/L in women. Bilateral fundus photography was performed and DR was diagnosed by clinical manifestations of vascular abnormalities in the retina including microaneurysms, retinal hemorrhages, hard exudates or vitreous hemorrhage. Stroke was defined as subarachnoid hemorrhage, cerebral venous thrombosis, spinal cord stroke and ischemic stroke. CHD was defined as having a history of angina with abnormal electrocardiogram or on stress test, myocardial infarction, angina coronary artery bypass graft surgery or angioplasty.

### 2.4 CKD definition

The new formula (22) for Chinese patients with type 2 diabetes was used to estimate glomerular filtration rate (eGFR) in milliliters per minute per 1.73m^2^, the formulas are: 313 × (Age)^-0.494^ × [Scr]^-1.059^ (mg/dl) × [Alb] ^0.485^ (g/dl) for men, and 783 × (Age)^- 0.489^ × [Scr]^-0.877^ (mg/dl) × [SUN]^-0.150^ (mg/dl) for women. In this analysis, CKD was defined as eGFR <60 mL/min/1.73m^2^ with or without kidney damage (23).

### 2.5 Measurement of amino acids

The details of the quantification of the amino acids assessment method were mentioned in a previous article (24). Briefly, mass spectrometry (MS) technology was applied to the metabolomics measurement. We collected capillary whole blood after a fast of at least 8 hours and stored it as dried blood spots (DBS) for metabolomic analysis. Metabolites in DBS were measured by direct infusion MS technology equipped with AB Sciex 4000 Qtrap system (AB Sciex, Framingham, MA, USA). High-purity water and Acetonitrile from Thermo Fisher (Waltham, MA, USA) were used as diluting agent and mobile phase.1-Butanol and acetyl chloride from Sigma-Aldrich (St Louis, MO, USA) was used to derive samples. Isotope-labeled internal standard samples of 12 amino acids (NSK-A) were purchased from Cambridge Isotope Laboratories (Tewksbury, MA, USA) while standard samples of the amino acids were purchased from Chrom systems (Grafelfing, Germany).

### 2.6 Statistical analysis

For continuous variables, normally distributed data were presented as mean ± SD (standard deviation), and skewed variables were expressed as median (IQR). Normality was tested by checking the Q-Q plot. Qualitative variables were expressed as numbers (percentage). Non-paired Student t-test (or Mann-Whitney U test if appropriate) in continuous data and Chi-square test (or fisher test when appropriate) in categorical variables were used to compare the difference between CKD and non-CKD. Arginine, ornithine, citrulline and the ratios of any two of them were classified into three categories based on the 33rd and 66th percentiles, respectively. Binary logistic regression models were performed to obtain odds ratios (ORs) and their 95% confidence intervals (CIs) of amino acids and the ratios of any two of them for the risk of CKD in univariate and multivariate analyses. A structured adjustment scheme was established to adjust for the effect of traditional risk factors on T2DM patients with CKD. We obtained the unadjusted OR values and the multivariable OR values adjusted for age, gender, BMI, duration of diabetes, systolic blood pressure (SBP), diastolic blood pressure (DBP), HDL-C, LDL-C, TG, HbA1c, smoking, drinking, anti-diabetes drugs, lipid-lowering drugs and antihypertensive drugs use.

Sensitivity analysis was performed to examine the consistency of the results in a 1032 population included patients with missing scream creatinine and serum albumin. A two-sided *P*<0.05 in all analyses was considered statistically significant. SAS version 9.4 (SAS institute Inc., Cary, NC, USA) was used to conduct the statistical analysis.

## 3 Results

### 3.1 Characteristics of study subjects

The characteristics of the study patients are shown in **Table 1**. The patients had a mean age of 58.0 (SD: 13.8) years and a mean duration of T2DM of 5 (0-10) years. The mean BMI of the cohort was 25.3 (SD: 3.8) kg/m^2^, with 44.5% of them being overweight and 21.0% being obese. Of the 855 patients, 52.3% were male, 143 were with CKD. The percentages of those with CHD, stroke and DR were 187 (21.9%), 181 (21.2%), and 115 (13.5%), respectively. The subjects with CKD were older and had a longer duration of diabetes, higher SBP, lower DBP, worse clinical profile in HbA1c, HDL-C, LDL-C and TG. These patients were more likely to be female and less likely to smoke and drink. Also, these patients were more likely to use β-blockers use and less likely to use metformin. Nevertheless, patients with CKD were higher rates of CHD as compared with those without CKD. HDL-C, triglyceride, HbA1c and use of drugs other than metformin and β-blockers were similar in the two groups.

**Table 1:** Clinical and biochemical characteristics of T2DM according to CKD.

Variables	Total (n=855)	CKD (n=143)	Non-CKD (n=712)	*P*-value
age	58.0 ± 13.8	63.9 ± 14.4	56.9 ± 13.4	<0.001
Duration of diabetes, years	5(0-10)	8(2-13)	5(0-10)	0.001
Male Gender	447(52.3%)	56(39.2%)	391(54.92%)	<0.001
BMI, kg/m^2^	25.3 ± 3.8	24.8 ± 4.1	25.4 ± 3.8	0.078
BMI < 18.5		8(5.6%)	14(2.0%)	0.025
BMI ≥18.5 and < 24		54(37.8%)	230(32.3%)	
BMI ≥24 and < 28		51(44.5%)	317(44.5%)	
BMI ≥28		30(21.0%)	151(21.2%)	
Smoke, yes		35(24.5%)	235(33.0%)	0.045
Drink, yes		24(16.8%)	206(28.9%)	0.003
SBP, mmHg		144.7 ± 25.7	139.8 ± 24.0	0.030
DBP, mmHg		78.7 ± 13.6	82.3 ± 13.3	<0.001
HDL-C, mmol/L		1.0(0.8-1.2)	1.0(0.9-1.3)	0.215
<1.00 in men or <1.30 in women		76(53.2%)	401(56.3%)	<0.001
≥1.00 in men or ≥1.30 in women		24(16.8%)	209(29.4%)	
Unknown		43(14.3%)	102(14.3%)	
LDL-C, mmol/L		2.6(2.1-3.1)	2.8(2.3-3.4)	0.027
<2.60		48(33.6%)	243(34.1%)	<0.001
≥2.60		52(36.4%)	367(51.5%)	
Unknown		43(30.1%)	102(14.3%)	
Triglyceride, mmol/L		1.7(1.1-2.2)	1.7(1.1-2.4)	0.718
<1.70		52(36.4%)	316(44.4%)	<0.001
≥1.70		48(33.6%)	296(41.6%)	
Unknown		43(30.1%)	100(14.0%)	
HbA1c, %		9.3 ± 2.5	9.6 ± 2.3	0.322
<7		18(12.6%)	54(7.6%)	0.014
≥7 and <8		13(9.1%)	84(11.8%)	
≥8		55(38.5%)	355(49.9%)	
Unknown		57(39.9%)	219(30.8%)	
Antidiabetic agents		122(85.3%)	592(83.2%)	0.524
Insulin		110(76.9%)	520(73.0%)	0.335
Metformin		32(22.4%)	265(37.2%)	<0.001
Other-OHD		61(42.7%)	303(42.6%)	0.982
Hypotensive agents		69(48.3%)	276(38.8%)	0.035
β-blockers		24(17.5%)	60(8.4%)	0.001
ACEI		24(16.8%)	89(12.5%)	0.168
ARB		20(14.0%)	96(13.5%)	0.873
Lipid-lowering agents		50(35.0%)	273(38.3%)	0.447
Statins		46(32.2%)	266(37.4%)	0.239
Other lipid-lowering agents		4(2.8%)	11(1.4%)	0.489
Coronary heart disease	187(21.9%)	47(32.9%)	140(19.7%)	<0.001
Stroke	181(21.2%)	38(26.6%)	143(20.1%)	0.083
Diabetic retinopathy	115(13.5%)	22(15.4%)	93(13.1%)	0.458

Data are presented as means ± SD, median (interquartile range), or n (%).

P value was acquired by comparing CKD and non-CKD; p values were derived from independent-samples Student t-test for normally distributed variables, Mann-Whitney U test for skewed distributions, and χ2 test (or Fisher test, if appropriate) for categorical variables.

BMI, body mass index; SBP, systolic blood pressure; DBP, diastolic blood pressure; HDL-C, high-density lipoprotein cholesterol; LDL-C, low-density lipoprotein cholesterol; HbA1c, glycated hemoglobin; Other-OHD, other-oral hypoglycemic drugs; ACEI, ACE inhibitor; ARB, angiotensin receptor blocker; T2DM, type 2 diabetes mellitus; CKD, chronic kidney disease.

The profile of serum amino acids related with urea cycle was shown in **Table 2**. Serum levels of citrulline, arginine and the ratios of arginine to ornithine and ornithine to citrulline were significantly different between the two groups. Compared with patients without CKD, the serum concentrations of citrulline and arginine were significantly higher in patients with CKD than those without. Ornithine was similar between the two groups. Moreover, the ratio of arginine to ornithine was markedly higher and the ratio of ornithine to citrulline was significantly lower in CKD than in those without CKD. Amino acids and their ratios were further stratified into tertiles, we observed that citrulline, the ratios of arginine to ornithine and ornithine to citrulline remained significant in the two comparison groups (all *p* values <0.05).

**Table 2:** Amino acids profile by CKD status.

Variables	CKD b(n=143)	Non-CKD (n=712)	*P*-value
Arg, μmol/L	12.6(7.0-17.9)	9.6(5.4-17.1)	0.030
Arg <6.86, μmol/L	35(24.5%)	247(34.7%)	0.059
Arg≥6.86 and <14.69 μmol/L	54(37.8%)	227(31.9%)	
Arg≥14.69, μmol/L	54(37.8%)	238(33.4%)	
Cit, μmol/L	22.9(18.1-32.5)	19.2(15.3-24.9)	<0.001
Cit<16.99, μmol/L	28(19.6%)	254(35.7%)	<0.001
Cit≥16.99 and <23.19, μmol/L	45(31.5%)	237(33.3%)	
Cit≥23.19, μmol/L	70(49.0%)	221(31.0%)	
Orn, μmol/L	17.4(12.8-23.6)	17.4(13.0-23.7)	0.767
Orn<14.31, μmol/L	46(32.2%)	235(33.0%)	0.762
Orn≥14.31 and <20.98, μmol/L	51(36.6%)	232(32.6%)	
Orn≥20.98, μmol/L	46(32.2%)	245(34.4%)	
Arg:orn	0.7(0.4-1.2)	0.6(0.3-1.0)	0.014
Arg:orn <0.42	33(23.0%)	252(35.4%)	0.012
Arg:orn≥0.42 and <0.85	50(35.0%)	229(32.2%)	
Arg:orn≥0.85	60(42.0%)	231(32.4%)	
Cit:arg	2.0(1.3-3.2)	1.9(1.2-3.3)	0.372
Cit:arg <1.38	44(30.8%)	237(33.3%)	0.705
Cit:arg≥1.38 and <2.70	52(36.4%)	234(32.9%)	
Cit:arg≥2.70	47(32.9%)	241(33.9%)	
Orn:cit	0.7(0.5-1.0)	0.9(0.7-1.2)	<0.001
Orn:cit <0.71	66(46.2%)	217(30.5%)	<0.001
Orn:cit≥0.71 and <1.04	46(32.2%)	234(32.9%)	
Orn:cit≥1.04	31(21.7%)	261(36.7%)	

Arg, arginine; Cit, citrulline; Orn, ornithine; Arg:orn, the ratio of arginine to ornithine; Cit:arg, the ratio of citrulline to arginine; Orn:cit, the ratio of ornithine to citrulline.

### 3.2 Associations of amino acids and their ratios with CKD risk

Citrulline, the ratio of ornithine to citrulline and arginine to ornithine were associated with the risk of CKD in analysis. As shown in **Table 3**, the top tertile of serum of citrulline in univariate analysis was associated with markedly increased risk of CKD as compared with its bottom tertile (OR: 2.87 [95%CI, 1.79-4.62]). After adjusting for possible confounders, i.e., age, gender, BMI, duration of T2DM, SBP, DBP, LDL-C, HDL-C, TG, HbA1c, drink, smoke, metformin, lipid-lowering drugs and β-blockers, in multivariable model 3, high levels of citrulline remained associated with significantly increased risk of CKD (OR: 2.38 [95%CI, 1.42-4.00]). In both univariate and multivariable analysis, the ORs of bottom vs top tertiles of the ratio of ornithine to citrulline for CKD were 2.56 (1.61-4.07) and 2.24 (1.29-3.87), respectively, with all ORs remaining statistically significant (**Table 4**). For the ratio of arginine to ornithine, 3rd tertile increased the risk of CKD compared with 1st tertile in univariate analysis (OR:1.98 [95%CI, 1.25-3.14]). Adjustmennt for traditional factors in multivariable model 3, the top tertile of the ratio of arginine to ornithine was also associated with increased risk of CKD as compared with its bottom tertile (OR: 2.14 [95%CI, 1.28-3.57]). However, in multivariable model 4, further adjustment for the ornithine to citrulline ratio, the ratio of arginine to ornithine was no longer significant for the risk of CKD (**Table 5**).

**Table 3:** Odds ratios of cit for the risk of CKD.

	OR (95% CI)	*P*-value	*P* for trend
Univariable model			
Cit<16.99, μmol/L	reference		<0.001
Cit≥16.99 and <23.19, μmol/L	1.72(1.04-2.85)	0.034	
Cit≥23.19, μmol/L	2.87(1.79-4.62)	<0.001	
Multivariable model 1			
Cit<16.99, μmol/L	reference		<0.001
Cit≥16.99 and <23.19, μmol/L	1.61(0.95-2.72)	0.078	
Cit≥23.19, μmol/L	2.50(1.52-4.14)	<0.001	
Multivariable model 2			
Cit<16.99, μmol/L	reference		<0.001
Cit≥16.99 and <23.19, μmol/L	1.52(0.89-2.58)	0.128	
Cit≥23.19, μmol/L	2.49(1.50-4.15)	<0.001	
Multivariable model 3			
Cit<16.99, μmol/L	reference		<0.001
Cit≥16.99 and <23.19, μmol/L	1.39(0.80-2.40)	0.246	
Cit≥23.19, μmol/L	2.38(1.42-4.00)	0.001	

Cit, citrulline.

Model 1 adjusted for age, gender, BMI and duration of T2DM.

Model 2 adjusted for age, gender, BMI, duration of T2DM, systolic blood pressure, diastolic blood pressure, high-density lipoprotein cholesterol, low-density lipoprotein cholesterol, triglyceride and HbA1c.

Model 3 adjusted for age, gender, BMI, duration of T2DM, systolic blood pressure, diastolic blood pressure, high-density lipoprotein cholesterol, low-density lipoprotein cholesterol, triglyceride, HbA1c, drink, smoke, metformin, lipid-lowering drugs and β-blockers.

**Table 4:** Odds ratios of orn:cit for the risk of CKD.

	OR (95% CI)	*P*-value	*P* for trend
Univariable model			
Orn:cit <0.71	2.56 (1.61-4.07)	<0.001	<0.001
Orn:cit≥0.71 and <1.04	1.66 (1.02-2.70)	0.043	
Orn:cit≥1.04	reference		
Multivariable model 1			
Orn:cit <0.71	2.25 (1.39-3.65)	0.001	<0.001
Orn:cit≥0.71 and <1.04	1.50 (0.90-2.49)	0.119	
Orn:cit≥1.04	reference		
Multivariable model 2			
Orn:cit <0.71	2.68 (1.61-4.44)	<0.001	<0.001
Orn:cit≥0.71 and <1.04	1.60 (0.95-2.71)	0.081	
Orn:cit≥1.04	reference		
Multivariable model 3			
Orn:cit <0.71	2.59 (1.55-4.35)	<0.001	<0.001
Orn:cit≥0.71 and <1.04	1.50 (0.88-2.58)	0.140	
Orn:cit≥1.04	reference		
Multivariable model 4			
Orn:cit <0.71	2.24 (1.29-3.87)	0.004	0.003
Orn:cit≥0.71 and <1.04	1.34 (0.77-2.33)	0.302	
Orn:cit≥1.04	reference		

Orn:cit, the ratio of ornithine to citrulline.

Model 1 adjusted for age, gender, BMI and duration of T2DM.

Model 2 adjusted for age, gender, BMI, duration of T2DM, systolic blood pressure, diastolic blood pressure high-density lipoprotein cholesterol, low-density lipoprotein cholesterol, triglyceride and HbA1c.

Model 3 adjusted for age, gender, BMI, duration of T2DM, systolic blood pressure, diastolic blood pressure, high-density lipoprotein cholesterol, low-density lipoprotein cholesterol, triglyceride, HbA1c, drink, smoke, metformin, lipid-lowering drugs and β-blockers.

Model 4 adjusted for age, gender, BMI, duration of T2DM, systolic blood pressure, diastolic blood pressure, high-density lipoprotein cholesterol, low-density lipoprotein cholesterol, triglyceride, HbA1c, drink, smoke, metformin, lipid-lowering drugs, β-blockers and the ratio of arginine to ornithine.

**Table 5:** Odds ratios of arg:orn for the risk of CKD.

	OR (95% CI)	*P*-value	*P* for trend
Univariable model			
Arg:orn <0.42	reference		0.004
Arg:orn≥0.42 and <0.85	1.67 (1.04-2.68)	0.035	
Arg:orn≥0.85	1.98 (1.25-3.14)	0.004	
Multivariable model 1			
Arg:orn <0.42	reference		0.002
Arg:orn≥0.42 and <0.85	1.84 (1.13-3.01)	0.015	
Arg:orn≥0.85	2.15 (1.32-3.48)	0.002	
Multivariable model 2			
Arg:orn <0.42	reference		0.004
Arg:orn≥0.42 and <0.85	1.72 (1.03-2.86)	0.038	
Arg:orn≥0.85	2.13 (1.29-3.51)	0.003	
Multivariable model 3			
Arg:orn <0.42	reference		0.004
Arg:orn≥0.42 and <0.85	1.65 (0.98-2.77)	0.061	
Arg:orn≥0.85	2.14 (1.28-3.57)	0.004	
Multivariable model 4			
Arg:orn <0.42	reference		0.115
Arg:orn≥0.42 and <0.85	1.48 (0.88-2.49)	0.145	
Arg:orn≥0.85	1.56 (0.91-2.68)	0.106	

Arg:orn, the ratio of arginine to ornithine.

Model 1 adjusted for age, gender, BMI and duration of T2DM.

Model 2 adjusted for age, gender, BMI, duration of T2DM, systolic blood pressure, diastolic blood pressure high-density lipoprotein cholesterol, low-density lipoprotein cholesterol, triglyceride and HbA1c.

Model 3 adjusted for age, gender, BMI, duration of T2DM, systolic blood pressure, diastolic blood pressure, high-density lipoprotein cholesterol, low-density lipoprotein cholesterol, triglyceride, HbA1c, drink, smoke, metformin, lipid-lowering drugs and β-blockers.

Model 4 adjusted for age, gender, BMI, duration of T2DM, systolic blood pressure, diastolic blood pressure, high-density lipoprotein cholesterol, low-density lipoprotein cholesterol, triglyceride, HbA1c, drink, smoke, metformin, lipid-lowering drugs, β-blockers and the ratio of ornithine to citrulline.

### 3.3 Sensitivity analysis

After inclusion the 177 study subjects with missing creatinine information and serum albumin information in the analysis, the effect sizes of citrulline and the ratio of ornithine to citrulline for CKD increased slightly and remained significant in univariable and multivariable analyses. (**Supplementary Tables 1**, **2**).

## Discussion

In this cross-sectional survey, we found that a higher concentration of citrulline was positively associated with increased risk of CKD in T2DM. The ratio of ornithine to citrulline was also associated with a markedly decreased risk of CKD in T2DM.

The progression of CKD increased the risk of all-cause mortality in T2DM patients (4, 25). Unfortunately, CKD in T2DM develops silently until severe damage has occurred. It is therefore crucial to find biomarkers that can detect decreased kidney function early within T2DM. The pathogenesis of CKD is complex and multifactorial, age, gender and duration of T2DM were typically unmodifiable risk factors for CKD; glycemic control, hypertension, lipid abnormalities, smoking and physical activity were modifiable risk factors for CKD (26). Insufficient insulin secretion was one of the risk factor of diabetic nephropathy (27); 5-methoxytryptophan (5-MTP), acetylcarnitine, taurine and tiglylcarnitine, were strongly correlated with the development of CKD (28). A prospective cohort study found that blood pressure levels, diabetes status, serum lipid status, obesity, smoking, and alcohol consumption affected the development of CKD (29). However, these markers are not sufficient to fully understand the pathogenesis of CKD, especially in T2DM. In our current study, we found that citrulline significantly increased the risk of CKD in T2DM. Some previous studies observed that citrulline was associated with diabetes. Research performed by Zhou Yong et al. revealed that plasma citrulline levels elevated in diabetes (30). A population-based study showed that plasma citrulline levels correlated with HbA1c levels (31). An animal experiment concluded that plasma citrulline and ornithine levels were elevated in obese and insulin-deficient mice, and further suggested that citrulline could be an early indicator of obesity-dependent metabolic impairment (32). Some encouraging data showed that citrulline could be a candidate predictor of renal injury, which was consistent with our findings. A GC-MS study showed that urinary metabolites differed between individuals with and without CKD in diabetes (33). A previou experiment comprising twelve mice showed that citrulline and arginine levels were elevated in CKD mice (16). Results derived from Framingham Heart Study (FHS) demonstrated that citrulline increased the risk of CKD (OR:1.48; 95%CI:1.19–1.83), indicating that the elevation of citrulline level in plasma might be a signal of underlying renal dysfunction (18). The results of an eight-year follow-up study from Korea showed that high levels of citrulline were strongly associated with the development of CKD (34). In addition, there were several studies documented that citrulline was associated with CKD progression or decreasing eGFR (17, 35, 36).

Interestingly, our study also found that the ornithine/citrulline ratio was reduced in CKD, which was in line with the results of some researchers. A study conducted in France in 2014 reported that citrulline/ornithine ratio increased in the late stages of CKD (36). A population-based study from South Africa showed that citrulline/ornithine ratio was significantly higher in the diabetic group compared to the non-diabetic group in stage 1 of CKD, indicating that this ratio may predict early CKD (37). It is worth noting that a small case-control study found that the ratio of citrulline to ornithine elevated in T2DM (38).

Citrulline is a non-essential amino acid, synthesized mainly in the intestine by the conversion of glutamine (39, 40). In kidney, the enzyme dimethyl arginine dimethyl amino hydrolase (DDAH) metabolizes asymmetric dimethylarginine (ADMA) to generate dimethylamine and citrulline, and subsequently citrulline is converted to arginine, a process that requires catalysis by argininosuccinate synthase (ASS) and argininosuccinate lyase (ASL) (16, 41). This could, to some extent, explain our findings that renal injury probably inhibits the activity of ASS or ASL, thereby affecting the conversion of citrulline to arginine (*de novo* synthesis of arginine) and resulting in abnormal arginine metabolism.

As we have described previously, citrulline and ornithine are two intermediates in arginine metabolism and an abnormal ratio of these two amino acids may reflect abnormal arginine metabolism. Abnormal ADMA metabolism is a manifestation of disorders of arginine metabolism. ADMA is produced by methylation of arginine residues in intracellular proteins by protein arginine N-methyltransferases (PRMTs), and over the past decades ADMA has been revealed to have biological properties that inhibit nitric oxide (NO) synthase, reduce NO bioavailability and lead to endothelial dysfunction (42, 43). NO is a potent endothelial vasodilator that balances the constrictor and regulates vascular tone and blood pressure. Some findings showed that plasma ADMA levels increased in the early stage of CKD (44–47) and that ADMA was negatively associated with eGFR (48, 49). Moreover, two cohort studies conducted in patients with CKD noted that ADMA favors CKD progression and renal function decline (50, 51). ADMA, therefore, explains well many pathophysiological aspects of CKD. We speculate that elevated plasma citrulline levels or abnormal ratio of ornithine to citrulline may be a consequence of the massive accumulation of ADMA in the vivo, which inhibits the synthesis of NO, a deficiency of which is a major feature of CKD.

There are two plausible conjectures regarding the results of our study: (1) renal injury interferes with arginine metabolism, resulting in abnormal citrulline and the ratio of ornithine to citrulline; (2) impaired citrulline metabolism may limit the bioavailability of arginine for nitric oxide synthesis, leading to endothelial dysfunction affecting renal function. Either explanation, however, previous studies were consistent in suggesting that citrulline or the ratio of ornithine to citrulline was closely associated with renal function. Our study drew the same conclusion: in patients with CKD, citrulline levels were significantly elevated and the ratio of ornithine to citrulline decreased. Large cohort studies and animal experiments are needed to validate the actual link between these metabolites and CKD.

Our research had critical implications for both public health and clinical practice. We found that patients with CKD had higher levels of citrulline and lower levels of ornithine/citrulline ratio. Our current study discovered an association between amino acids related to the urea cycle and CKD, which dramatically widens our appreciation of pathological mechanisms of CKD. Furthermore, our findings shed light on the prevention of CKD and the delay of its progression.

There were several limitations in our current study. First, because our study was a retrospective cross-sectional investigation, the true causality could not be validated. However, our findings were consistent with some earlier studies, which might support our results. Second, given the nature of cross-sectional study, only statistical associations between citrulline, the ratio of ornithine to citrulline and CKD could be revealed, not causal relationships. Diabetes was not only accompanied by aberrations in amino acid metabolism, but also accelerated kidney damage, and we cannot determine whether urea cycle disturbances resulted from CKD in our study. These findings need to be confirmed in prospective cohort studies. Third, our study involved T2DM patients who were hospitalized and likely had more severe CKD and T2DM. Therefore, it is important to be cautious when extending our results to other populations. Forth, LDL-C, HDL-C, TG, and HbA1c had a large number of missing values. Taking into account that amino acids related to urea cycle rather than glucose and lipids were the main factors in this study, we regarded the missing values as a category. Finally, diet may affect citrulline levels (52), however, information on diet was not collected and not available to the analysis.

In conclusion, we found that high serum citrulline was significantly associated with increased risk of CKD and ornithine/citrulline ratio was inversely associated with risk of CKD in T2DM. Prospective cohort studies from different populations with T2DM are required to replicate our findings and mechanistic investigations are also warranted to understand molecular mechanisms underlying the biological link between serum amino acids involved in urea cycle and risk of CKD.

## Data availability statement

The original contributions presented in the study are included in the article/**Supplementary Material**. Further inquiries can be directed to the corresponding author/s.

## Ethics statement

The studies involving human participants were reviewed and approved by First Affiliated Hospital of Liaoning Medical University. The ethics committee waived the requirement of written informed consent for participation. Written informed consent was not obtained from the individual(s) for the publication of any potentially identifiable images or data included in this article.

## Author contributions

QZ and Z-ZF conceived the project, designed experiments. WZ wrote the manuscript and analyzed data. JuZ collected the information and contributed to the writing of this manuscript. XY conceived the project, designed experiments and interpretated the data. NL contributed to the data interpretation and data analysis. JiZ collect the information and contributed to the data interpretation and wrote the manuscript and analyzed data. All authors edited the final version of the manuscript. All authors contributed to the article and approved the submitted version.
