# Supplementary material for: Associations of serum amino acids related to urea cycle with risk of chronic kidney disease in Chinese with type 2 diabetes
Source: Front Endocrinol (Lausanne). 2023 Mar 2;14:1117308. doi: 10.3389/fendo.2023.1117308 (PMC10018121; doi:10.3389/fendo.2023.1117308)
Supplement: Supplementary file 1 [file DataSheet_1.zip › Supplementary Table 1.DOCX]

Supplementary Material

**Associations of serum amino acids related to urea cycle with risk of chronic kidney disease in Chinese with type 2 diabetes**

Wei Zhang^1,#^, Jun Zheng^2,#^, Jikun Zhang^3^, Ninghua Li^1^, Xilin Yang^1^, Zhong-Ze Fang ^3,*^ Qiang Zhang^2,*^

# These two authors equally contributed to this work

^1^Department of Epidemiology and Biostatistics, School of Public Health, Tianjin Medical University, Tianjin, China;

^2^Department of Geriatrics, Tianjin Medical University General Hospital, Tianjin Geriatrics Institute, Tianjin, China;

^3^Department of Toxicology and Sanitary Chemistry, School of Public Health, Tianjin Medical University, Tianjin, China

***Correspondence and Reprint Addressed to:** Qiang Zhang, Department of Geriatrics, Tianjin Medical University General Hospital, Tianjin Geriatrics Institute, Tianjin 300052, China; Tel: +86-22-60363665; E-mail: zhangqiangyulv@163.com

or

Prof. Zhongze Fang, Department of Toxicology, School of Public Health, Tianjin Medical University, 22 Qixiangtai Road, Heping District, Tianjin 300070, China;

Tel: +86 22 83336637; Email: [fangzhongze@tmu.edu.cn](mailto:fangzhongze@tmu.edu.cn)

Table 1 Odds ratios of cit for the risk of CKD

|  | OR（95% CI） | *P*-value | *P* for trend |
| --- | --- | --- | --- |
| Univariable model |  |  |  |
| Cit<16.81, mol/L | reference |  | <0.001 |
| Cit≥16.81 and <23.16, mol/L | 1.31（0.72-2.36） | 0.376 |  |
| Cit≥23.16, mol/L | 3.45（2.06-5.79） | <0.001 |  |
| Multivariable model 1 |  |  |  |
| Cit<16.81, mol/L | reference |  | <0.001 |
| Cit≥16.81 and <23.16, mol/L | 1.13（0.60-2.12） | 0.699 |  |
| Cit≥23.16, mol/L | 2.55（1.46-4.45） | 0.001 |  |
| Multivariable model 2 |  |  |  |
| Cit<16.81, mol/L | reference |  | <0.001 |
| Cit≥16.81 and <23.16, mol/L | 1.08（0.57-2.05） | 0.805 |  |
| Cit≥23.16, mol/L | 2.42（1.39-4.26） | 0.002 |  |
| Multivariable model 3 |  |  |  |
| Cit<16.81, mol/L | reference |  | <0.001 |
| Cit≥16.81 and <23.16, mol/L | 0.99（0.52-1.92） | 0.998 |  |
| Cit≥23.16, mol/L | 2.32（1.30-4.13） | 0.004 |  |

Abbreviations: Cit, citrulline

Model 1 adjusted for age, gender, BMI and duration of T2DM

Model 2 adjusted for age, gender, BMI, duration of T2DM, systolic blood pressure, high-density lipoprotein cholesterol, low-density lipoprotein cholesterol, triglyceride and HbA1c

Model 3 adjusted for age, gender, BMI, duration of T2DM, systolic blood pressure, diastolic blood pressure, high-density lipoprotein cholesterol, low-density lipoprotein cholesterol, triglyceride, HbA1c, drink, smoke, metformin, lipid-lowering drugs and β-blockers

Model 4 adjusted for age, gender, BMI, duration of T2DM, systolic blood pressure, diastolic blood pressure, high-density lipoprotein cholesterol, low-density lipoprotein cholesterol, triglyceride, HbA1c, drink, smoke, metformin, lipid-lowering drugs, β-blockers and arginine

Table 2 Odds ratios of orn:cit for the risk of CKD

|  | OR（95% CI） | *P*-value | *P* for trend |
| --- | --- | --- | --- |
| Univariable model |  |  |  |
| Orn:cit <0.71 | 4.19（2.35-7.46） | <0.001 | <0.001 |
| Orn:cit≥0.71 and <1.05 | 2.80（1.54-5.09） | <0.001 |  |
| Orn:cit≥1.05 | reference |  |  |
| Multivariable model 1 |  |  |  |
| Orn:cit <0.71 | 3.22（1.75-5.91） | <0.001 | <0.001 |
| Orn:cit≥0.71 and <1.05 | 2.29（1.22-4.31） | 0.010 |  |
| Orn:cit≥1.05 | reference |  |  |
| Multivariable model 2 |  |  |  |
| Orn:cit <0.71 | 3.47（1.86-6.48） | <0.001 | <0.001 |
| Orn:cit≥0.71 and <1.05 | 2.22（1.16-4.25） | 0.016 |  |
| Orn:cit≥1.05 | reference |  |  |
| Multivariable model 3 |  |  |  |
| Orn:cit <0.71 | 3.60（1.89-6.87） | <0.001 | <0.001 |
| Orn:cit≥0.71 and <1.05 | 2.14（1.10-4.18） | 0.025 |  |
| Orn:cit≥1.05 | reference |  |  |
| Multivariable model 4 |  |  |  |
| Orn:cit <0.71 | 3.49（1.75-6.97） | <0.001 | <0.001 |
| Orn:cit≥0.71 and <1.05 | 1.99（1.00-3.98） | 0.049 |  |
| Orn:cit≥1.05 | reference |  |  |

Abbreviations: Orn:cit, the ratio of ornithine to citrulline

Model 1 adjusted for age, gender, BMI and duration of T2DM

Model 2 adjusted for age, gender, BMI, duration of T2DM, systolic blood pressure, high-density lipoprotein cholesterol, low-density lipoprotein cholesterol, triglyceride and HbA1c

Model 3 adjusted for age, gender, BMI, duration of T2DM, systolic blood pressure, diastolic blood pressure, high-density lipoprotein cholesterol, low-density lipoprotein cholesterol, triglyceride, HbA1c, drink, smoke, metformin, lipid-lowering drugs and β-blockers

Model 4 adjusted for age, gender, BMI, duration of T2DM, systolic blood pressure, diastolic blood pressure, high-density lipoprotein cholesterol, low-density lipoprotein cholesterol, triglyceride, HbA1c, drink, smoke, metformin, lipid-lowering drugs, β-blockers and the ratio of arginine to ornithine
